# Bedside and continuous assessment of arterial load in critically-ill patients by combined analysis of esophageal doppler blood flow and arterial pressure waveform: preliminary results

**DOI:** 10.1186/2197-425X-3-S1-A593

**Published:** 2015-10-01

**Authors:** MI Monge García, A Santos Oviedo, P Guijo González, M Gracia Romero, A Gil Cano

**Affiliations:** Hospital SAS de Jerez de la Frontera, Unidad de Cuidados Intensivos, Jerez de la Frontera, Spain; Massachusetts General Hospital, Department of Anesthesia, Boston, MA USA; Uppsala University, Surgical Sciences Department, Uppsala, Sweden; M+Vision Cofund, Madrid, Spain

## Introduction

Because the oscillatory nature of the arterial pressure and blood flow, the input impedance assessed in frequency domain provides the best description of the arterial load [1]. However, this approach is complex and not feasible in clinical practice. Nowadays, it is possible to measure simultaneously flow and pressure at the bedside using minimally invasive monitoring.

## Objectives

To test the usefulness of continuous assessment of arterial load based on a 3-element Windkessel model by a combined analysis of the esophageal Doppler derived-blood flow and arterial pressure waveform, against standard frequency-domain analysis of arterial impedance.

## Methods

**Figure 1:**
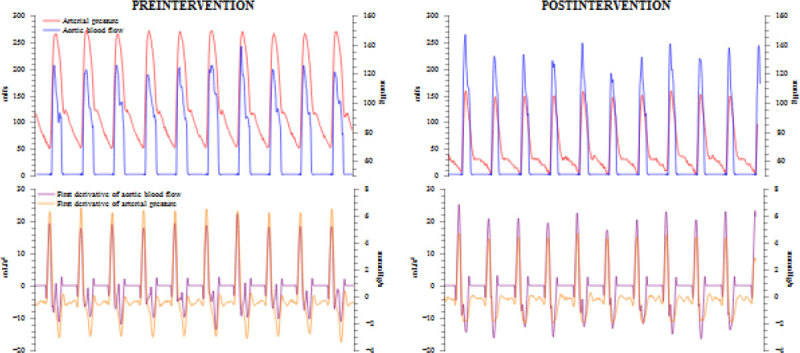
Figure 1

**Figure 2:**
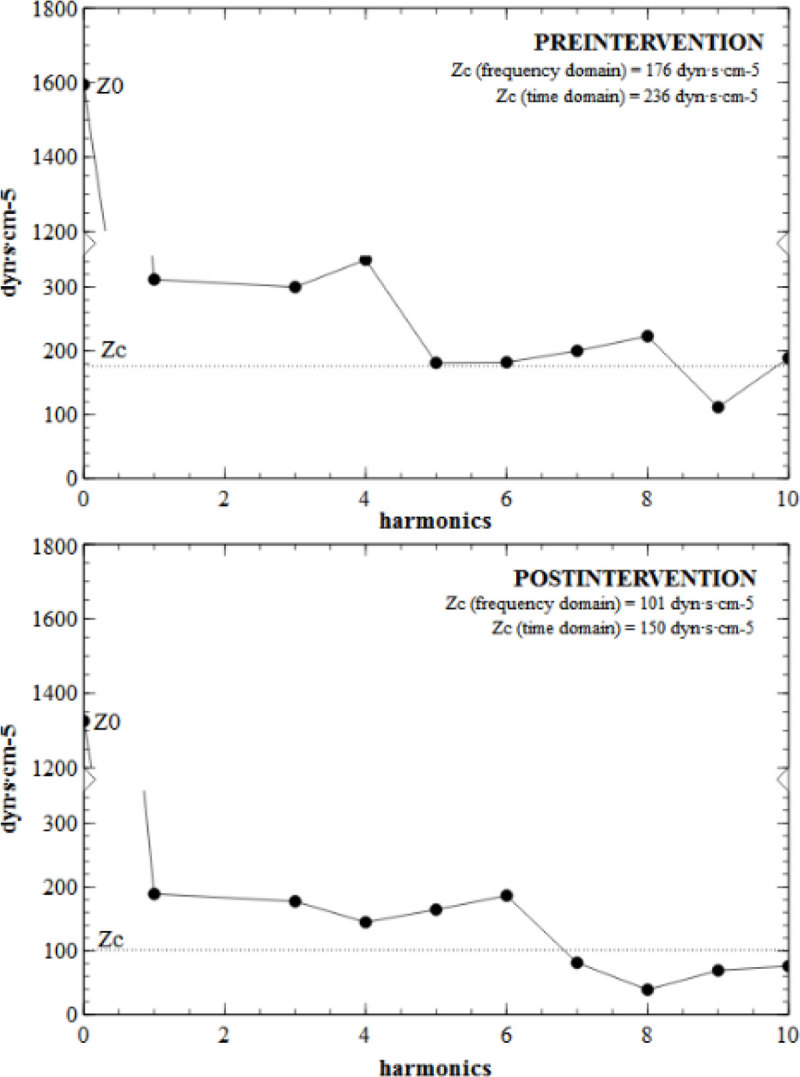
Figure 2

Time-domain variables of arterial impedance were obtained by the simultaneous analysis of the Doppler derived-blood flow and arterial pressure. A 3-element Windkessel model was used, consisting on arterial resistance (R = mean arterial pressure/cardiac output), arterial compliance [C = stroke volume (SV)/arterial pulse pressure), characteristic impedance [Zc_t_ = maximum derivative of pressure (dP_max_/dt)/maximum derivative of aortic blood flow (dQ_max_/dt)] (Figure 1). Effective arterial elastance (Ea = 90% of systolic arterial pressure/SV) was used as a lumped parameter of the whole arterial load. Frequency-domain analysis of the pressure-blood flow waveforms was obtained to estimate the input impedance (Z_in_), characteristic impedance (Zc, average of the modulus of 4^th^ and 10^th^ harmonics, Figure 2), and Z_1_ (modulus at the first harmonic, as an index of compliance). Reflection coefficient (RC) in time and frequency domain was estimated as (SVR-Zc)/(SVR+Zc). Measurements were obtained in patients in whom a vasodilators or vasoconstrictor was introduced or dosage was changed, and a significant variation in arterial load was expected.

## Results

Ten measurements in 6 patients were obtained. Time and frequency-domain derived Zc were similar at pre(222 vs 203 dyn•s•cm^-5^; P = n.s.) and post-intervention (205 vs 165 dyn•s•cm^-5^; P = n.s.). Pre and post-intervention values and percentage changes of Zc and Zc_t_ were strongly correlated (R^2^ = 0.94, R^2^ = 0.95; R^2^ = 0.96; P < 0.0001). Time and frequency-domain derived reflection coefficient were also correlated at pre (R^2^ = 0.93; P < 0.0001), post-intervention (R^2^ = 0.85; P < 0.0001) and percentage changes (R^2^ = 0.85; P < 0.0001). Changes in net arterial compliance and Z_1_ were also inversely correlated (R^2^ = 0.59; P < 0.0001). As expected, Z_0_ and R showed an equivalent behavior.

## Conclusions

Our preliminary results shows that continuous and noninvasive assessment of different aspects of arterial load by combined analysis of Doppler derived-aortic blood flow and arterial pressure could be feasible at the bedside and comparable to standard frequency domain analysis.
